# A single alcohol binge impacts on neutrophil function without changes in gut barrier function and gut microbiome composition in healthy volunteers

**DOI:** 10.1371/journal.pone.0211703

**Published:** 2019-02-01

**Authors:** Vanessa Stadlbauer, Angela Horvath, Irina Komarova, Bianca Schmerboeck, Nicole Feldbacher, Sonja Wurm, Ingeborg Klymiuk, Marija Durdevic, Florian Rainer, Andreas Blesl, Sarah Stryeck, Tobias Madl, Philipp Stiegler, Bettina Leber

**Affiliations:** 1 Department of Gastroenterology and Hepatology, Medical University of Graz, Graz, Austria; 2 Center for Biomarker Research in Medicine (CBmed), Graz, Austria; 3 Center for Medical Research, Core Facility Molecular Biology, Medical University of Graz, Graz, Austria; 4 Core Facility Computational Bioanalytics, Medical University of Graz, Graz, Austria; 5 Gottfried Schatz Research Center for Cell Signaling, Metabolism and Aging, Molecular Biology and Biochemistry, Medical University of Graz, Graz, Graz, Austria; 6 BioTechMed, Graz, Austria; 7 Department of Transplantation Surgery, Medical University of Graz, Graz, Austria; University Hospital Oldenburg, GERMANY

## Abstract

Alcohol binge drinking is a dangerous drinking habit, associated with neurological problems and inflammation. The impact of a single alcohol binge on innate immunity, gut barrier and gut microbiome was studied. In this cohort study 15 healthy volunteers received 2 ml vodka 40% v/v ethanol/kg body weight. Neutrophil function was studied by flow cytometry; markers of gut permeability and inflammation (lactulose/mannitol/sucrose test, zonulin, calprotectin, diamino-oxidase) were studied with NMR spectroscopy and enzyme-linked immunosorbent assay in urine, stool and serum respectively. Bacterial products in serum were quantified using different reporter cell lines. Gut microbiome composition was studied by 16S rDNA sequencing and bioinformatics analysis. After a single alcohol binge, neutrophils were transiently primed and the response to *E*.*coli* stimulation with reactive oxygen species (ROS) production was transiently increased, on the other hand the percentage of neutrophils that did not perform phagocytosis increased. No changes in gut permeability, inflammatory biomarker, bacterial translocation and microbiome composition could be detected up to 4 hours after a single alcohol binge or on the next day. A single alcohol binge in young, healthy volunteers transiently impacts on neutrophil function. Although the exact biological consequence of this finding is not clear yet, we believe that this strengthens the importance to avoid any alcohol binge drinking, even in young, otherwise healthy persons.

## Introduction

Alcohol binge drinking, defined as 5 or more drinks for men and 4 or more drinks for women within 2 hours, is the most frequent form of alcohol consumption worldwide, especially in younger people [1]. This drinking pattern is popular and leads to increased mortality and morbidity. Therefore, binge drinking is a major public health issue. The behavioral and neurological consequences of binge drinking are well characterized [2–4]. Less is known about the systemic effects on the intestine as the first organ in contact with alcohol and on the consequences on inflammation and immune function. Chronic alcohol intake can lead to increased gut permeability, bacterial translocation and alterations in the gut microbiome in animal models [5–7]. Recently bacterial translocation has been shown in healthy volunteers after a single alcohol binge [8] and in frequent binge drinkers [9]. On immune cells, alcohol intake seems to have dichotomous effects. On the one hand alcohol-induced liver injury is driven by pro-inflammatory reactions, whereas in the long-term, immunosuppressive and anti-inflammatory effects have been described [10]. These immune effects, via Toll-like receptors, may be driven by endotoxin or other bacterial products that are translocated to the circulation via a defective gut barrier [11]. It is so far unknown what effects a single alcohol binge has on gut microbiome, gut barrier, bacterial translocation and innate immunity. We therefore aimed to study whether a single alcohol binge with 2 ml of vodka 40% v/v ethanol/kg body weight impairs gut barrier and gut microbiome composition, increases bacterial translocation and inflammation and impacts on neutrophil dysfunction.

## Methods

### Participants

From January 2017 to March 2017 we conducted an observational pilot study at the Clinical Research Center, Medical University of Graz in volunteers (>21 years old, willing to abstain from alcohol 48h prior to the study visits). The study was conducted according to the Declaration of Helsinki, was approved by the institutional review board of the Medical University of Graz (28–255 ex 15/16) and registered at clinicaltrials.gov (NCT02568904). Volunteers were invited from an institutional database and gave written informed consent. We excluded subjects with known alcohol abuse (Alcohol Use Disorders Identification Test ≥ 8 in men or ≥ 7 in women or CAGE test ≥ 2 for both men and women), elevated liver function test or with any disease or medication that does not allow concomitant consumption of alcohol. We ruled out pregnancy and lactation in women. After informed consent and overnight fasting, participants received 2 ml of vodka 40% v/v ethanol/kg body weight in a total volume of 300 ml orange or strawberry juice within 30 minutes at the Clinical research center. The drink was prepared by the study personnel. Blood samples were taken from a venous line at baseline and hourly up to 4h after the first sip of alcohol for neutrophil function and serum markers of gut permeability, inflammation and bacterial translocation. One day before and on the day after the alcohol binge participants performed a lactulose/mannitol/sucrose test for gut permeability and collected a stool sample. Participants received a payment of 100€ upon completion of the study. The study was designed as pilot study to assess the magnitude of effects of one binge drink; therefore no formal sample size calculation was performed.

### Innate immune function

Phagoburst and Phagotest kits (Glycotope, Heidelberg, Germany) were used as per manufacturer’s instruction to determine activation and burst profiles of neutrophils by flow cytometric analysis with modifications in the analysis strategy as previously described by our group [12]. To determine oxidative burst function, the production of reactive oxygen species of neutrophils after incubation with (a) saline solution (resting burst), (b) N-Formylmethionine-leucyl-phenylalanine (priming) or (c) *E*. *coli* (oxidative burst) is assessed, as instructed by the manufacturer. After red blood cell lysis and fixation of the cells, flow cytometic analysis was done. The neutrophil population was gated in the forward-side scatter plot and 10.000 gated cells were recorded. In a next step, the main population of the cells incubated in saline solution (resting burst) was used to establish a patient specific threshold. Cells with a geometric median fluorescence intensity (GMFI) exceeding this threshold were considered positive (i.e. producing reactive oxygen species). Resting burst, priming and oxidative burst were defined as the amount of positive cells after incubation with the accoding stimulus (a-c).

Phagocytosis was measured utilizing FITC-labelled *E*. *coli* that were added to two samples of whole blood per patient. One sample was incubated for 10 minutes at 37°C while the other sample remained on ice. After the incubation, extracellular FITC-signals were quenched (reagent provided in the assay kit). After red blood cell lysis and fixation of the cells, flow cytometry analysis was done. Ten thousand neutrophils were recorded and analysed. The main population of the sample kept on ice was used to establish a patient specific positivity threshold. Non-phagocytizing neutrophils were defined as cells not exceeding this threshold.

### Gut permeability, inflammation and bacterial translocation

Enzyme linked immunosorbent assays (ELISA) were used to measure fecal and serum calprotectin, fecal and serum zonulin, serum diamine oxidase (Immundiagnostik AG, Bensheim, Germany), soluble (s)CD14 (R&D Systems, Minneapolis, USA), and lipopolysaccharide binding protein (LBP) (Hycult Biotech, Uden, The Netherlands). All assays were performed according to manufacturers’ instructions.

For the lactulose/mannitol/sucrose test, after overnight fasting, the study participants drank a solution of 100 ml water containing 10g lactulose, 5g D-mannitol and 20g sucrose. Urine is collected over 5 hours. After 2 hours consumption of water is allowed. Sugar recovery in urine test was analyzed by NMR spectroscopy as published previously [12].

Bacterial products (endotoxin, peptidoglycan and bacterial DNA) were detected in serum using HEK-Blue hTLR4, HEK-Blue hNOD2 and HEK-Blue hTLR9 reporter cells (Invivogen, Toulouse, France), respectively as published previously [12].

### Microbiome analysis

Total DNA was isolated from frozen stool samples using MagnaPure LC DNA Isolation Kit III (Bacteria, Fungi) (Roche, Mannheim, Germany) according to manufacturer’s instructions including mechanic and enzymatic lysis [13]. Hypervariable regions V1-2 were amplified in a target specific PCR using the primers 27F and R357 (27F-AGAGTTTGATCCTGGCTCAG; R357-CTGCTGCCTYCCGTA) and sequenced with the Illumina MiSeq technique (Illumina, Eindhoven, The Netherlands) [13]. Sequencing was done in cooperation with the Core Facility for Molecular Biology at the Center for Medical Research in Graz.

### Statistical analysis

For microbiome analysis generated FASTQ files were prepared for analysis using Qiime tools [14] implemented in Galaxy (https://galaxy.medunigraz.at) [15]. High quality reads were clustered based on 97% similarity. Unfiltered OTUs rarefied to 19741 reads per sample were used for alpha diversity assessment with Chao1 index and low abundance filter with 0.005 minimum count fraction and at least two different hosts were applied before beta diversity analysis. Principal component analysis was based on Bray Curtis dissimilarity. For OTU abundance comparisons Mann-Whitney-U-tests were performed. Benjamini-Hochberg procedure was used for microbiome data to account for multiple testing. PICRUSt [16] implemented in Galaxy was used to predict Kyoto Encyclopedia of Genes and Genomes (KEGG) functional pathway abundance content from 16S rDNA data using a closed reference OTU table created in QIIME and the Greengenes reference database—version 13 8. Sequence data are made available at the NCBI sequence read archive (SRP143475).

All other statistical analyses were performed using SPSS version 23.0 (SPSS Inc., Chicago, Illinois, USA) and GraphPad Prism version 7.00 for Windows (GraphPad Software, California, USA) for visualizations. Tests were chosen depending on the distribution of the data assessed by D´Agostino-Pearson normality test and the number of groups to compare. Multiple comparisons were done by one-way ANOVA, Kruskal-Wallis or Friedman test with either the Holm-Sidak method or Dunn´s method as post-hoc test depending on the type of data. All statistical tests were 2-sided, and p-values <0.05 were considered statistically significant.

## Results

**Fig 1:**
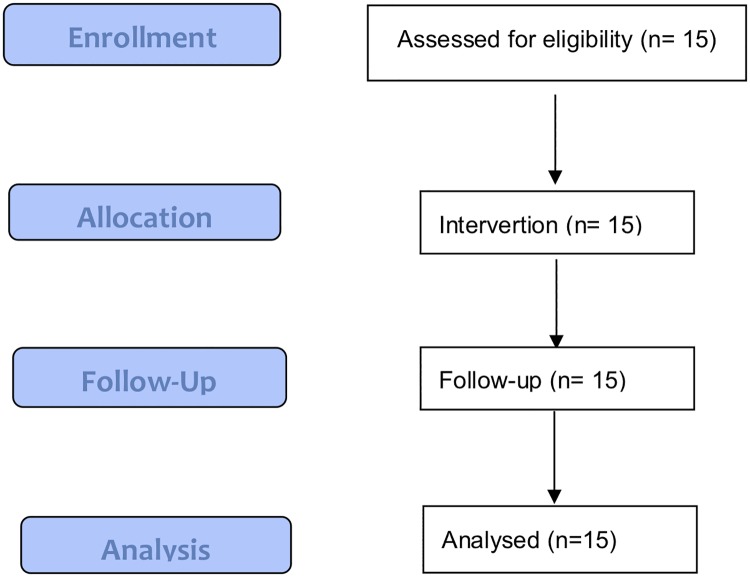
Study flowchart.

15 volunteers participated in the study and all individuals completed the study (Fig 1). Baseline characteristics are given in Table 1. None of the volunteers had any evidence of acute or chronic diseases at the time of the study and no drug (prescription or non-perceptional) intake was reported. Alcohol binge did not cause any severe adverse events. Smoking status did not have any impact on any parameters at baseline.

**Table 1:** Baseline characteristics.

Parameter	N = 15
**Age (years, mean ± SD)**	26±4
**Gender (m/f)**	11/4
**Smoking (y/n)**	10/5
**BMI (kg/m**^**2**^**)**	24.5 (23.0; 27.2)
**Sport (h per week)**	5.5 (2.5; 10)
**Drinks (per week)**	2 (1; 3)
**AST (U/L)**	25 (19;37)
**ALT (U/L)**	23 (15;36)
**Creatinine (mg/dL)**	0.95 (0.79; 0.97)

BMI: Body mass index; AST Aspartate-Aminotransferase, ALT: Alanine-Aminotransferase, Data are given as median and interquartile range unless indicated otherwise

**Fig 2:**
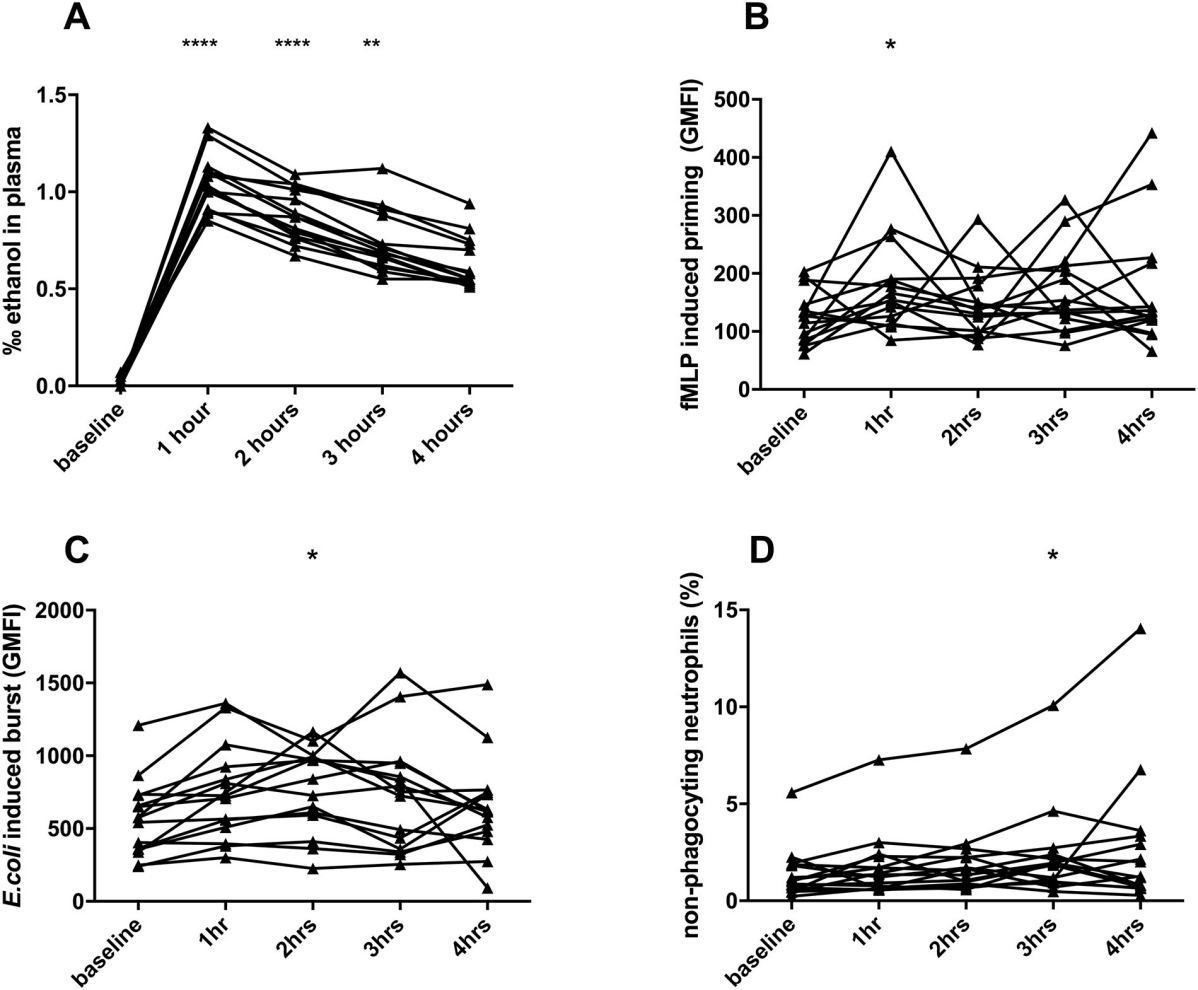
(A) Blood alcohol levels, (B) priming of neutrophils (ROS production after stimulation with N-formyl-methionyl-leucyl-phenylalanine (fMLP)), (C) neutrophil ROS production after stimulation with *E*.*coli* (D) percentage of non-phagocytosing neutrophils after exposure with FITC-labelled *E*.*coli*. Measurements were performed before and after a single alcohol binge over 4 hours. *p<0.05, **p<0.01, ****p<0.0001, Friedman test with Dunns multiple comparison test.

Blood alcohol levels peaked 1 hour after intake and gradually decreased thereafter (Fig 2A). One hour after alcohol binge neutrophils showed significantly more ROS production after stimulation with N-formyl-methionyl-leucyl-phenylalanine (fMLP), indicating that they were readier to respond to further stimuli (priming) (Fig 2B). ROS production after *E*. *coli* stimulation increased and reached statistical significance compared to baseline 2h after alcohol binge (Fig 2C). The number of neutrophils that did not show phagocytosis of FITC labelled *E*.*coli* bacteria increased slightly, but significantly at 3h after alcohol binge compared to baseline. (Fig 2D) Representative FACS histograms are shown in S1 Fig. Smoking status did not have any impact on any parameters during the time course of the study.

**Fig 3:**
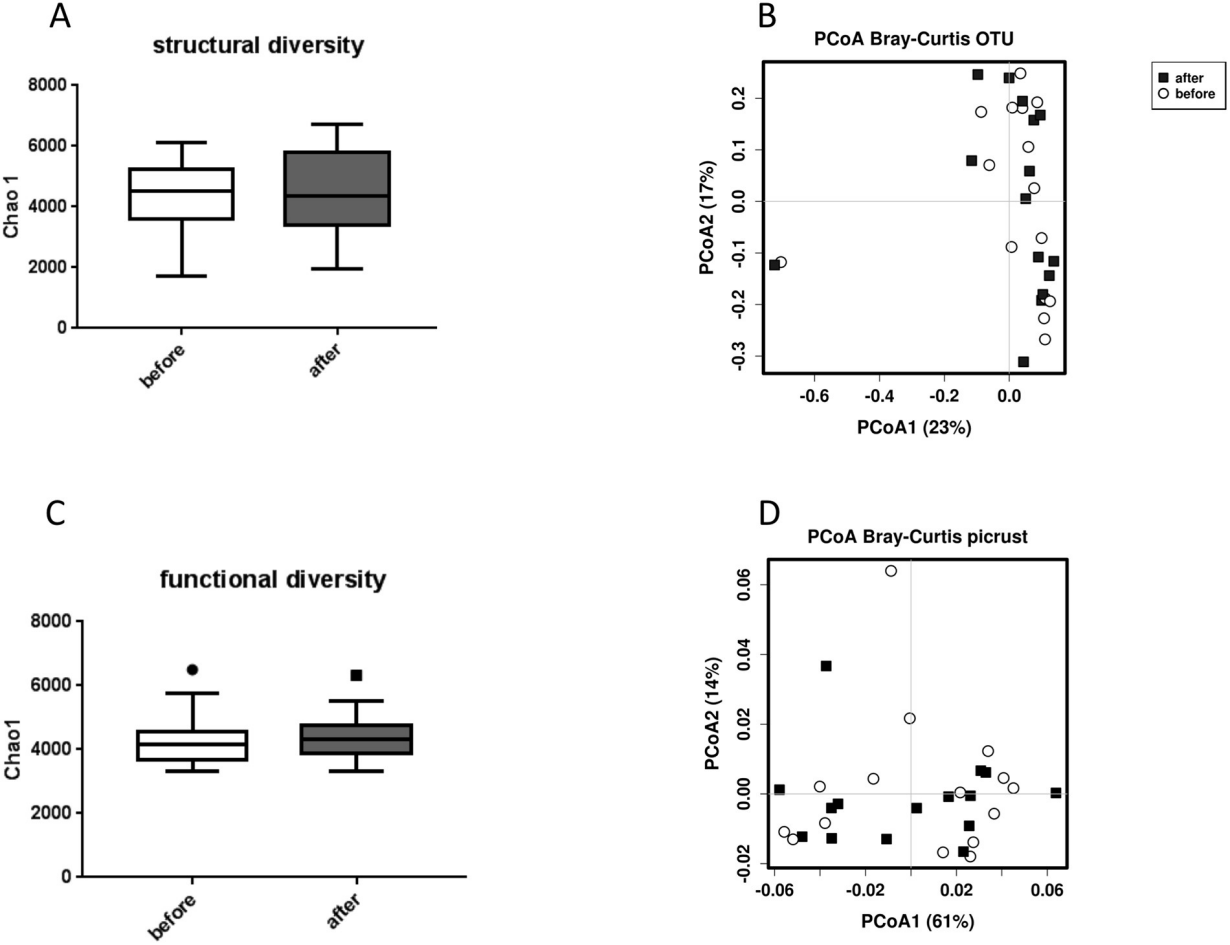
(A) Bacterial alpha diversity (Chao1) of the stool microbiome before and after alcohol binge, (B) PCoA plot of bacterial beta-diversity based on the Bray-Curtis dissimilarity (C) Alpha diversity (Chao1) of function content assessed by PICRUSt (D) PCoA plot of beta-diversity based on the Bray-Curtis dissimilarity of function content assessed by PICRUSt.

Alcohol binge did not impair gut permeability as measured by fecal zonulin (marker of tight junction integrity), lactulose/mannitol ratio (large intestinal permeability) and sucrose recovery (gastroduodenal permeability) before and after alcohol binge as well as by serum zonulin and diamino-oxidase in serum during the alcohol binge (Table 2). Also, calprotectin in stool as a marker of intestinal inflammation and calprotectin in serum as a marker of systemic inflammation did not change after a single alcohol binge (Table 2). We could not verify increased levels of endotoxin, endotoxin binding proteins, peptidoglycans or bacterial DNA in blood during our observation period of 4h after alcohol binge (Table 2). No changes in structural and functional composition of the stool microbiome could be detected by 16s sequencing. Alpha diversity and beta diversity did not differ between the two time points. (Fig 3A and 3B) No taxonomic group differences on phylum, class, order, family, genus and OTU level were detectable. Using predicted metagenome function by PICRUSt, functional alpha and beta diversity did not change and no group differences could be detected after a single alcohol binge. (Fig 3C and 3D) Smoking status did not have any impact on gut permeability inflammation bacterial translocation of microbiome composition or function.

**Table 2:** Gut permeability, bacterial translocation and microbial diversity before (1 day before for lactulose/mannitol, sucrose, fecal calprotectin, fecal zonulin and immediately before the binge for all serum markers), during 4h after alcohol binge (serum markers) and 1 day after alcohol binge (lactulose/mannitol, sucrose, fecal calprotectin, fecal zonulin).

	Before (n = 15)	1h after (n = 15)	2h after (n = 15)	3h after (n = 15)	4h after (n = 15)	1 day after (n = 15)
**Zonulin serum (ng/ml)**	47.8 (38.9; 70.1)	52.7 (40.6; 67.8)	53.6 (39.0; 67.4)	52.1(39.8; 61.4)	53.0 (42.2; 63.4)	-
**Calprotectin serum (ng/ml)**	598.7 (409.0; 1090)	773.7 (514.8; 1229)	629.3 (433.9; 820.7)	574.2 (357.6; 1108)	602.5 (386.3; 855.1)	-
**Diamino-oxidase (U/L)**	15.2 (9.7;25.3)	11.4 (8.3;22.0)	12.1 (9.3;21.0)	12.0 (9.2;17.2)	11.9 (8.4;20.0)	-
**Endotoxin (EU/ml)**	1.2 (1.0;1.9)	1.4 (0.6;1.6)	0.7(0.3;1.4)	0.7 (0.4;2.3)	0.8 (0.3;2.2)	-
**LBP (μg/ml)**	12.8 (10.6;16.3)	11.2 (8.6;12.9)	13.5 (9.8;14.6)	12.2 (8.9;16.2)	11.5 (8.6;14.8)	-
**sCD14 (μg/ml)**	1.9 (1.4;2.4)	1.7 (1.6;2.1)	1.7 (1.5;1.9)	1.7 (1.5;2.0)	1.7 (1.5;2.2)	-
**Peptidoglycan (ng/ml)**	0.8 (0.6;1.0)	0.7 (0.5; 0.9)	0.8 (0.5;0.9)	0.7 (0.6; 1.1)	0.7 (0.5; 0.8)	-
**Bacterial DNA (μM)**	14.5 (8.8; 16.1)	13.8 (9.9; 17.0)	14.5 (6.6; 16.6)	12.8? (5.5; 17.4)	11.8 (5.9, 17.4)	-
**Fecal zonulin (ng/ml)**	86.3 (56.0; 193.8)	-	-	-	-	107.9 (59.5; 154.3)
**Lactulose/mannitol ratio**	0 (0; 0.006)	-	-	-	-	0.003 (0: 0.009)
**Sucrose recovery (%)**	0.002 (0.0; 0.004)	-	-	-	-	0.004 (0.02; 0.07)
**Fecal calprotectin (ng/ml)**	31.5 (27.6;32.4)	-	-	-	-	31.5 (19.9; 43.6)

Data are presented as median (Q1; Q3), LBP: lipopolysaccharide binding protein, sCD14: soluble CD14

## Discussion

Our study shows that one single alcohol binge in young, healthy volunteers without dangerous alcohol consumption habits impacts on innate immune function, even though it has no detectable effect on gut permeability, bacterial translocation and microbiome composition.

Binge drinking is a common and increasing health problem. It is well described that repetitive binge drinking has detrimental effects on cognitive and social functioning [17]. Innate immune response to binge drinking impacts on neuroinflammation and may aggravate neural damage [18]. In animal models, ethanol increases TLR2 and TLR4 expression in the brain [19]. This leads to the hypothesis, that pathogen associated molecular patterns (PAMPs) may be involved in the pathogenesis of neural damage.

We could demonstrate that a single alcohol binge impacts on neutrophil function, causing increased priming and subsequently increased ROS production upon stimulation with *E*.*coli*, but also an increase of non-phagocytosing *E*.*coli*. The time course of the functional changes implies that alcohol might cause increased readiness to respond to further stimulation and due to increased ROS production, fewer cells are capable to perform phagocytosis as it has already been shown in vitro when incubating neutrophils with fluoride. [20] Although the changes in neutrophil function in our cohort of healthy volunteers were transient and mild, since neutrophil burst parameters returned to baseline at the end of the 4h observation period and non-phagocytosing neutrophils increased only slightly, there is evidence from animal models, that alcohol can hamper innate immune response. Single or short term alcohol binge impairs immune response to induced sepsis or burn injury, increases mortality in mouse models [21–23] and decreases the macrophage response [24]. In humans, clinically relevant concentrations of ethanol attenuate bactericidal activity of primed neutrophils, [25] although increased priming of neutrophils by other substances, such as endotoxin, should optimize other neutrophil functions. [26] Recently it has been proposed that ethanol diminishes neutrophil extracellular trap formation and killing of pathogens via a CXCL1 mediated pathway [27]. Besides those apparent direct negative effects of alcohol on innate immune function, ethanol at concentrations equivalent to serum concentrations found with binge drinking, can also improve bacterial biofilm formation, which might enhance pathogenicity of bacteria [24,28,29]. However, the design of our study does not allow correlation with clinically significant endpoints such as infections. Furthermore the small sample size and the physiological variation of neutrophil function in human studies limit the possibility to draw firm conclusions from these data. Unfortunately, due to ethical concerns regarding the effects of binge drinking in healthy volunteers, we could not increase the sample size.

We could not demonstrate increased bacterial translocation or changes in gut permeability, inflammation or microbiome composition in our study, despite studying a large panel of markers in blood, urine and stool. In a study in healthy volunteers, using a similar protocol to ours, a significant increase in endotoxin, bacterial DNA and endotoxin binding proteins was seen 1h after the alcohol binge [8]. The subjects in our study had a lower BMI and were younger than the cohort described by Bala et al [8]. Since, obesity and metabolic syndrome increases gut permeability and changes gut microbiome composition [30,31], it is likely that in the cohort studied by Bala et al. some subjects already had subtle changes in gut barrier function due to subclinical metabolic disorders and therefore a single alcohol binge aggravated barrier dysfunction and led to bacterial translocation into the peripheral circulation. This hypothesis is also supported by the finding, that alcohol and high fat diet both increase gut permeability and have a synergistic effect on hepatic inflammation [32]. Furthermore, we could not detect any effects of one single alcohol binge on gut microbiome structure and function. So far, no detailed studies on the time course of alcohol induced microbiome changes have been performed, therefore it was unclear whether a single alcohol binge causes changes in microbiome community structure or function. [33] While it has been clearly demonstrated that a diet change affects the gut microbiome community structure as fast as after 24 hours, [34] our data suggest that alcohol effects on the gut microbiome seem to take longer.

## Conclusion

Taken together, a single alcohol binge in young, healthy persons without any dangerous alcohol drinking behavior, impacts on neutrophil burst and phagocytic function, but gut microbiome composition and function and the gut barrier function remains unaltered. Innate immune dysfunction during long-term alcohol misuse therefore may not only be caused by bacterial products leading to “immune paralysis” [35] but also directly through ethanol effect on neutrophil granulocytes or through other indirect effects not related to the gut barrier.

## Supporting information

S1 Fig FACS histogram examplary. Histogramms for resting burst (yellow line), priming (blue line) and oxidative burst (red line)during a single alcohol binge.
(PDF)

S1 Checklist Trend checklist. Trend checklist for reporting of nonrandomized trials.
(PDF)

S1 Protocol Study protocol V3. The protocol version when the study reported herein was performed.
(DOCX)

S2 Protocol Study protocol V7. The most recent study protocol of this trial.
(PDF)
