# Supplementary material for: A single alcohol binge impacts on neutrophil function without changes in gut barrier function and gut microbiome composition in healthy volunteers
Source: PLoS One. 2019 Feb 1;14(2):e0211703. doi: 10.1371/journal.pone.0211703 (PMC6358085; doi:10.1371/journal.pone.0211703)
Supplement: S1 Fig — Histogramms for resting burst (yellow line), priming (blue line) and oxidative burst (red line)during a single alcohol binge. (PDF) [file pone.0211703.s001.pdf]

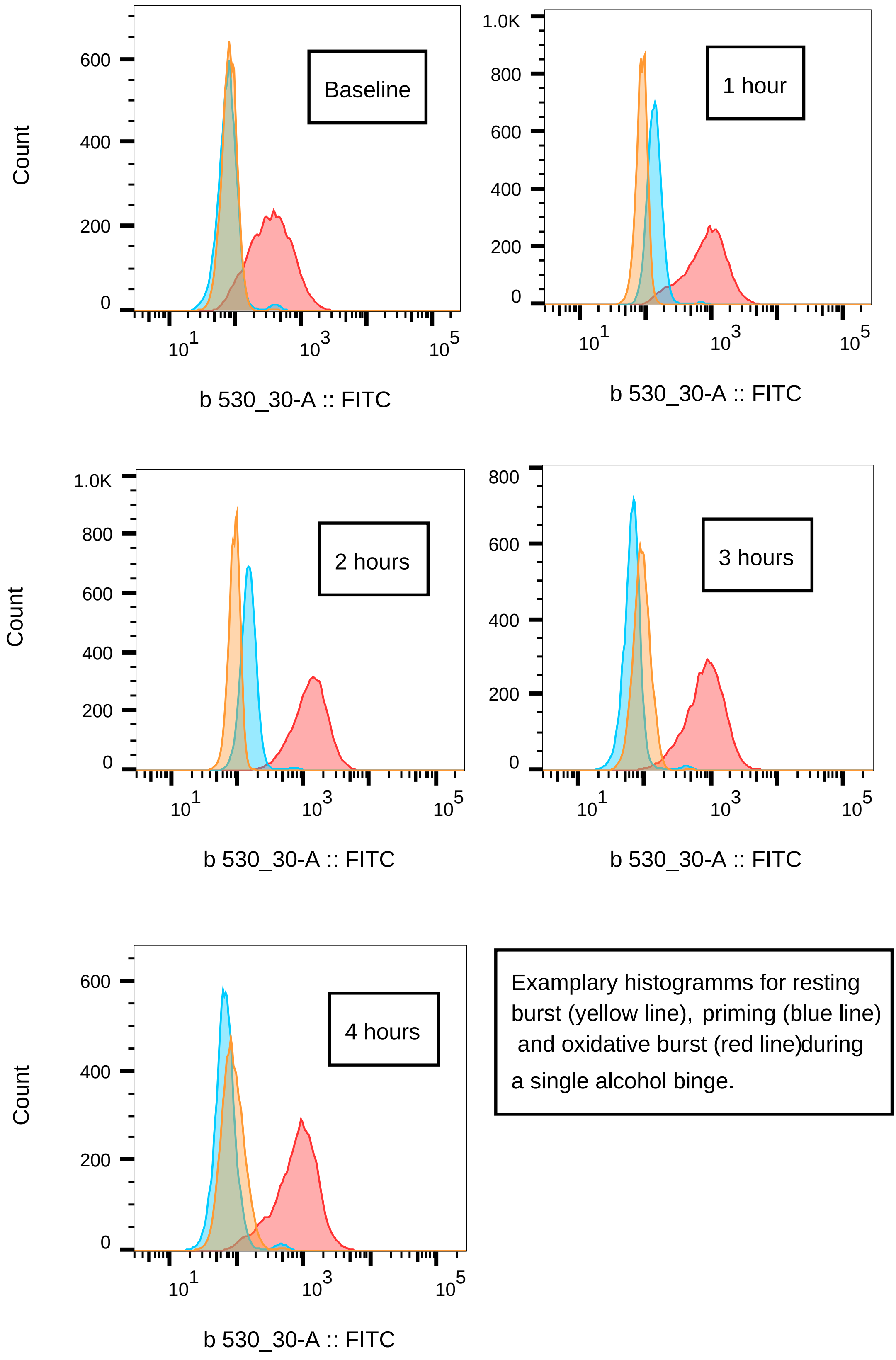

Examplary histogramms for resting burst (yellow line), priming (blue line) and oxidative burst (red line)during a single alcohol binge.
