# Supplementary material for: A single alcohol binge impacts on neutrophil function without changes in gut barrier function and gut microbiome composition in healthy volunteers
Source: PLoS One. 2019 Feb 1;14(2):e0211703. doi: 10.1371/journal.pone.0211703 (PMC6358085; doi:10.1371/journal.pone.0211703)
Supplement: S1 Protocol — The protocol version when the study reported herein was performed. (DOCX) [file pone.0211703.s003.docx]

**The effects of binge drinking on innate host defence mechanisms**

**Short title: Alcohol study**

**Study protocol**

**Version 3**

**03.10.2016**

**Principal Investigator**

*Assoz. Prof. Dr. Vanessa Stadlbauer-Köllner*

Department of Internal Medicine, Division of Gastroenterology and Hepatology, Medical University of Graz

**Study Team**

*Mag. Angela Horvath*

*Monika Tawdrous, Bsc*

*Dr. Bettina Leber*

*Bianca Schmerböck, Bsc*

**Cooperation partners**

*Dr. Ingeborg Klymiuk*

Core Facility Molecular Biology, Medical University of Graz

Table of Content

1 Background and state of the art 3

2 Hypothesis 3

3 Preliminary data 4

4 Study 6

4.1 Recruitment 6

5 Endpoints 6

6 Subject selection 6

6.1 Inclusion criteria 6

6.2 Exclusion criteria 7

7 Visit Schedule 7

8 Methods 10

8.1 Sample size 10

8.2 Tests 10

**8.2.1** **Gut permeability** 10

**8.2.2** **Bacterial products** 10

**8.2.3** **Inflammation** 12

**8.2.4** **Neutrophil function** 13

**8.2.5** **Statistical analysis** 13

9 Work and Time Plan 13

10 Ethical considerations 13

11 Quality control 14

12 Insurance 14

13 Responsibilities 14

14 References 15

#

# Background and state of the art

Alcohol binge drinking, defined as 5 or more drinks for men and 4 or more drinks for women at one time, is the most frequent form of alcohol consumption worldwide, especially in younger people. [1] This drinking pattern is popular and leads to increased mortality and morbidity. Therefore binge drinking is a major public health issue. The behavioural and neurological consequences of binge drinking are well characterized [2-4].

Less is known about the systemic effects on the gut as the first organ in contact with alcohol and on the consequences on inflammation and immune function. Chronic alcohol intake can lead to increased gut permeability, bacterial translocation and alterations in the gut microbiome in animal models. [5-7] Recently bacterial translocation has been shown in healthy volunteers after a single alcohol binge. [8] On immune cells, acute alcohol intake seems to have dichotomous effects. On the one hand alcohol induced liver injury is driven by pro-inflammatory reactions, whereas in the long-term, immunosuppressive and anti-inflammatory effects have been described. [9] These immune effects seem to be driven by endotoxin or other bacterial products via Toll-like receptors that are translocated to the circulation via a defective gut barrier. [10]

# Hypothesis

We hypothesize that binge drinking impairs gut barrier, increases bacterial translocation and causes inflammation. This leads to an early increase in neutrophil and monocyte function but also an increase in oxidative stress. (see Figure 1).


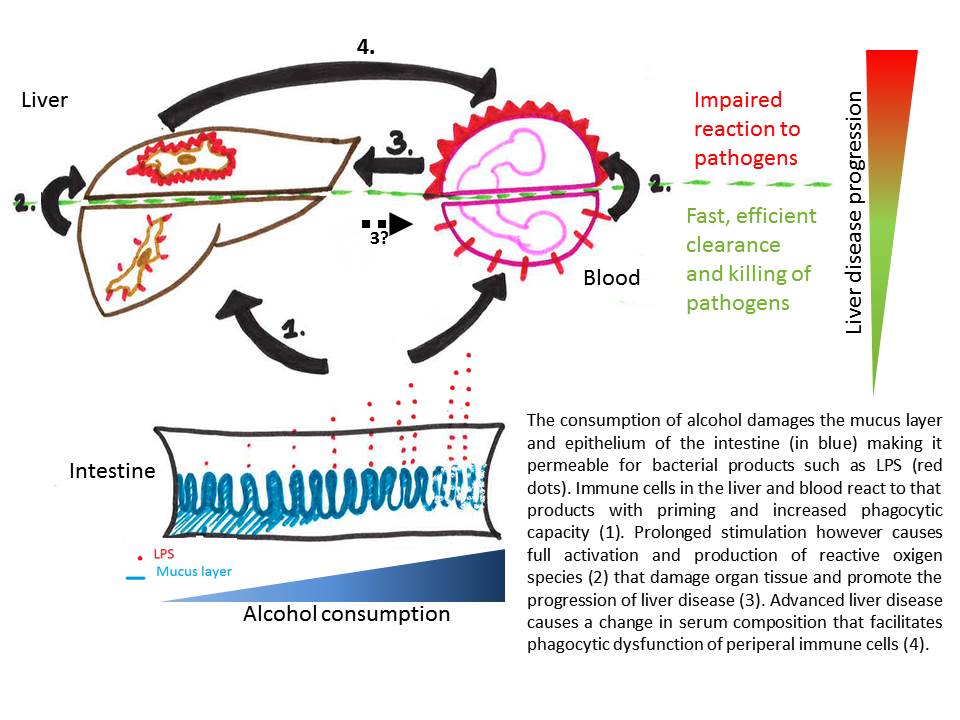


Figure 1: Hypothesis of how alcohol consumption impacts on gut permeability and liver/phagocyte function.

# Preliminary data

In previous research projects we gathered preliminary data that draw a crude picture of the influence that alcohol has on the innate immune system. In 55 abstinent patients with alcohol-induced liver cirrhosis we found that neutrophil function depends on liver synthesis. Patients with compensated cirrhosis (Child-Pugh grade A) showed a significant decrease in phagocytic activity (percentage of phagocytizing neutrophils) but a slight increase in phagocytic capacity (semi quantitative measurement of the amount of engulfed bacteria). It is possible that with the increase in inactive cells the remaining functioning cells compensate this loss by increasing their capacity. In decompensated cirrhosis (Child-Pugh grade B/C) this compensation seems to be lost and neutrophil dysfunction becomes apparent.

To clarify the role of liver damage in alcohol induced immune disorder we utilized the Lieber-DeCarli model of alcohol feeding on C57BL/6 mice that exhibit similar damage to the gut barrier and microbiome as humans do but only develop very mild alcoholic liver disease (ALD). We found a significantly increased *ex vivo* clearance capacity of whole blood, a trend to improved neutrophil function, as well as a significantly faster, more efficient clearance of pathogens from the blood in early stages of experimentally induced sepsis. When the livers of these mice were damaged with 3,5-diethoxycarbonyl-1,4-dihydrocollidine (DDC) before the alcohol feeding period, the before mentioned advantages in *ex vivo* function tests were lost.

We therefore, hypothesize that alcohol by itself has immune activating properties as long as the liver is still functional. However, once the liver has been damaged the effectiveness of the immune system decreases with the degree of liver disease.


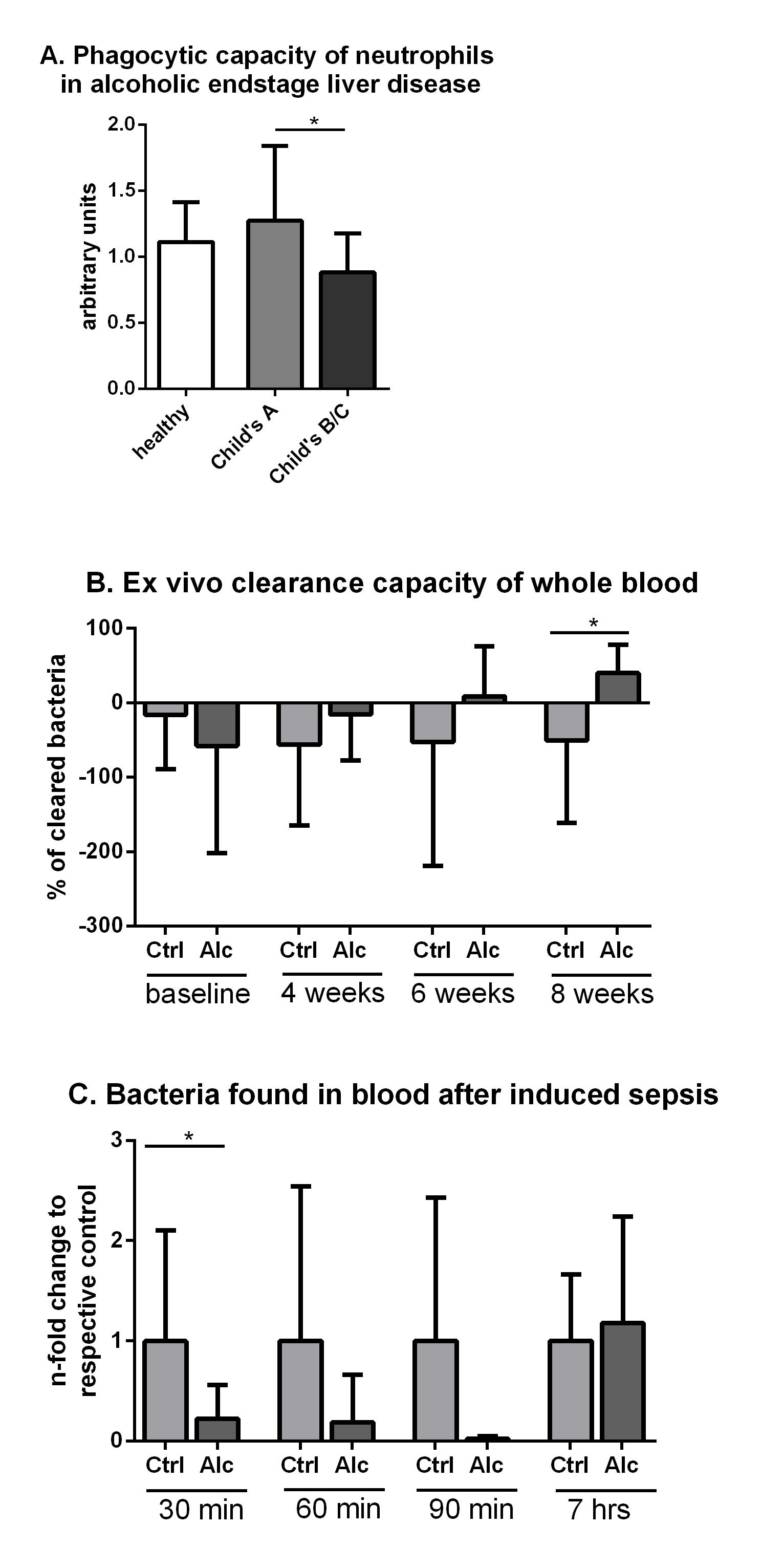


Fig. 2: A. Phagocytic capacity of neutrophils of 55 patients with alcoholic end stage liver disease (39 Child’s A and 16 Child’s B or C) compared to 30 healthy controls. Capacity is presented as GMFI weighted by the percentage of active cells, normalized to batch controls. B. Ex vivo clearance capacity of whole blood challenged with *E. coli* of 20 mice on Lieber-DeCarli alcohol or control diet. C. Bacterial load in the blood stream of 19 mice on Lieber-DeCarli alcohol or control diet 30, 60, 90 minutes and 7 hours after intravenous injection of *E. coli.*

Data presented as mean and SD, *significant on 5% level (or significant after Bonferroni correction for multiple comparison)

# Study

This study should test whether one episode of binge drinking in otherwise healthy volunteers causes bacterial translocation and innate immune activation.

## Recruitment

Healthy volunteers will be recruited from the volunteer database of the Clinical Research Center, Medical University Graz

# Endpoints

Primary endpoint

stool zonulin levels

Secondary endpoints

bacterial translocation

gut permeability

bacterial translocation

inflammation

innate immune function

gut microbiome

# Subject selection

## Inclusion criteria

- Participant is willing and able to give informed consent for participation in the study.
- Age above 18 years
- Willingness to abstain from alcohol 48h prior to the study visits

## Exclusion criteria

- Alcohol abuse
  - Alcohol Use Disorders Identification Test ≥ 8 in men or ≥ 7 in women

or

- - CAGE test ≥ 2 (both men and women)
- Elevated liver function test
- Any disease or medication that does not allow concomitant consumption of alcohol
- Women: pregnancy and lactation

# Visit Schedule

Volunteers will undergo 1 screening visit and 2 study visits.

The screening visit duration is approximately 1 hour, the first study visits will last at least 5 hours with an optional observation/recovery period thereafter until the alcohol breath test is below 0.5 ‰. On the next day patients will have to perform the gut permeability test at home and will deliver the urine sample together with a stool sample and a questionnaire to the trial centre.

**Table 1**

**Schedule of assessments**

| **Visit no.** | **1 (Screening)** | **2** | **3** |
| --- | --- | --- | --- |
| **Timing** | 0-28 days prior to visit 1a |  | 24h after visit 1a |
| Inclusion / exclusion criteria | X |  |  |
| Demographics/relevant medical history/current medical conditions | X | X | X |
| Physical examination | X | X |  |
| Standard laboratory tests | X | X |  |
| Lactulose/Mannitol/Sucrose Test | X |  | X |
| pregnancy test (in women of childbearing age) | X | X |  |
| Blood sampling for gut permeability, bacterial translocation, oxidative stress and innate immune function (50 ml) | X | X |  |
| Stool sampling | X |  | X |
| Dosage administration, adverse events, Concomitant medications/therapies | Ongoing data capture | | |

**Screening visit 1**

Male and female healthy volunteers who accomplish the inclusion criteria and none of the exclusion criteria are eligible for the study and will undergo the following investigation procedures in the screening visit, after signing the informed consent:

- Detailed explanation of the study procedures, including the intake of alcohol under medical supervision
- Check inclusion and exclusion criteria
- Check for demographic data, medical and surgical history and concomitant medication
- Date of birth, gender, smoking status, alcohol consumption
- Body weight and body height
- Vital signs: resting pulse and blood pressure
- Physical examination
- Pregnancy test in women of childbearing age.
- Blood sampling
- Randomization
- Distribution of the material for the gut permeability urine test and stool sampling

**Visit 2**

Subjects will be informed about the nature of the study, including the intake of alcohol. The patients have to sign that they will not use a car or a machine on the day of the study. They have to state how they will travel home after the study (either taxi, public transport or being picked up by an adult friend or family member).

As part of this visit the following procedures will be performed:

- Body weight
- Vital signs: resting pulse and blood pressure
- Physical examination
- Pregnancy test in women of childbearing age
- Alcohol breath test
- Blood sampling via a venous access line before alcohol consumption and every hour for 4 hours.
- Consumption of 2 ml vodka 40% v/v ethanol/kg body weight in a total volume of 300 ml orange or strawberry juice

Subjects will be monitored at the Clinical Research Center until the alcohol breath test is below 0.5 ‰. Subjects will be provided with a taxi voucher.

**Visit 3**

24h after visit 1 they patients will have to perform the gut permeability urine test for 5h at home, collect a stool sample and fill in the self-administered hangover questionnaire and will have to bring the urine sample, the stool sample and the questionnaire to the trial site.

# Methods

## Sample size

In order to determine the magnitude of effect on gut permeability and innate immune function by a single alcohol binge, we intend to perform a pilot study on 12 healthy volunteers. After these 12 subjects, the test parameters described below will be analysed and the magnitude of effect will be determined. These results will be the basis for a second study to study potential mechanisms to improve gut permeability and innate immune function.

## Tests

The assays described in this section are the current state of the art. It is possible that the methodology changes until analysis or that assays need to be exchanged for technical reasons.

### **Gut permeability**

A ready-to-use solid-phase sandwich ELISA (Immundiagnostik AG, Bensheim, Germany) is used to detect DAO, Zonulin and Calprotectin in serum and stool samples. The test is performed according to the manufacturer’s instructions.

Lactulose/Mannitol test: After overnight fasting, the patient drinks a solution of 100 ml water containing 10g lactulose, 1g D-mannitol and 20g sucrose. Urine is collected over 5 hours while fasting is continued for 3 hours after study start. The urine volume collected during the 5 hours is wrote down and 1ml aliquots are frozen immediately at -80°C with 10% of a 1% thimerosal solution for subsequent analysis by NMR spectroscopy.

### **Bacterial products**

*Endotoxin*

Endotoxin is detected by the HEK-blue LPS detection kit, a cell based colorimetric assay. HEK-blue-4 cells are very sensitive to LPS because of the expression of TLR4. TLR4 is a receptor recognizing the lipid moiety of LPS. In these engineered cells the binding of LPS leads to the activation of NF-κB, which then changes the colour of the detection medium. The quantification occurs by reading the OD at 650nm. HEK-cells are split when they reach a confluence of 80%. 5*10^4^ cells are added in each well of a 24-well plate and incubated at 37°C, 5% CO_2_ overnight. A standard dilution series and positive controls are prepared. The growth medium is exchanged by detection medium. Standards, controls and patient serum are added in triplicates and incubated overnight. On the next day 100µl supernatant is transferred in duplicates to a 96-well plate and the colour change is quantified photometrically (InvivoGen, USA)

Endotoxin binding proteins

A ready-to-use solid-phase sandwich ELISA (Hycult biotechnology, Uden, Netherlands) is used to detect LBP and sCD14 levels in EDTA plasma samples.

Bacterial DNA in serum

Circulating bacterial DNA from Plasma is extracted with the QIAamp Circulating Nucleic Acid Kit (Qiagen, Venlo, Netherlands) according to the manufacturer’s instructions. Bacterial 16S rDNA is amplified with the Mastermix 16s Complete PCR Kit (Molzym, Bremen, Germany) using primers with a template-specific sequence 16s_515_S3_fwd: GATTGCCAGCAGCCGCGGTAA and 16s_806_S2_rev: GGACTACCAGGGTATCTAAT. In a second PCR, fusion primers of 16s_515_S3_fwd with a sample specific nucleotide barcode sequence and A-Adapter and 16s_806_S2_rev with P-Adapters are used to allow for a simultaneous analysis of multiple samples on a semiconductor based sequencing run (Ion PGM Sequencer, Thermo Fisher Scientific, MA, USA). PCR products are separated on a 2% SeaKem ME agarose gel (Cambrex, Rockland, ME, USA) and specific bands (~ 350 bp) are excised and DNA extracted using the Qiagen gel extraction kit (Qiagen) and eluted in 30 µL of Aqua dest.

Amplicon DNA concentrations are determined using the Quant-iT PicoGreen dsDNA Assay Kit according to the manufacturer’s instructions. Sequencing is performed by using the Ion PGM Sequencer with an Ion Sequencing HiQ Kit (all Thermo Fisher Scientific, MA, USA). Raw reads are de-noised, filtered and identified using MOTHUR. Resulting high quality reads are analysed using QIIME. Alpha rarefaction compared with paired t-tests corrected with Benjamini-Hochberg False Discovery Rate procedure and Beta-diversity compared with ANOSIM serve as comparable parameters between groups.

### **Inflammation**

Plasma cytokines

Cytokines interleukin (IL)-6, IL-8, IL-10 and TNF-alpha are determined by flow cytometry. A ProcartaPlexTM four-plex-assay (eBioscience, Vienna, Austria) of these cytokines is performed according to the manufacturer’s instructions. Measurement is performed by a BioRad BioPlex200 multiplex suspension array system (Bio-Rad GesmbH, Vienna, Austria).A ready-to-use solid-phase sandwich ELISA (eBioscience, Vienna, Austria) is used to detect soluble IL-6 receptor levels in serum samples. The test is performed according to the manufacturer’s instructions. Measurement is performed on a SpectraMax 384 plate reader in combination with the SoftMax Pro 4.6 Software (Molecular Devices, Ismaning, Germany) at 450nm versus 620nm as reference wavelength.

Carbonyl protein (CP) ELISA

CP is measured by ELISA. To quantify the protein carbonyl content in samples protein carbonyls have to be derivatized with dinitrophenyl-hydrazine (DNPH). Carbonyls are detected and quantified via the DNPH anti-DNPH antibody complex (Immundiagnostik, Germany).

Advanced Oxidation Protein Products (AOPP)

Serum is depleted of ApoB-containing lipoproteins with polyetylenglycol (PEG). 400 μL PEG-solution (20% PEG in 200 mmol/L glycine, pH = 7.4) is added to 1 mL serum and incubates for 20 min. Precipitate is pelleted by centrifugation (10.000 x g, 30 min) and the supernatant is used for AOPP detection. Subsequently, 10 μL apoB-depleted serum is mixed with 40 μL 0.2 mol/L citrate buffer and incubated for 2 min on a shaker. Afterwards, absorbance at 340 nm was measured on a Nano Drop 1000 (Peqlab, Erlangen, Germany) spectrophotometer. Absorbance is converted into the respective AOPP concentrations by means of a standard curve ranging from 1 to 100 μmol/L chloramine-T.

### **Neutrophil function**

The Phagoburst® kit (Glycotope, Heidelberg, Germany) will be used to determine activation and burst profiles of neutrophils and monocytes by flow cytometric analysis. Cells will be discriminated forward-side-scatter characteristics. Phagocytosis will be assessed by Phagotest® (Glycotope, Heidelberg, Germany), a flow cytometric analysis using FITC-labelled *E. coli* bacteria.

### **Statistical analysis**

Data will be analyzed with SPSS 21. Data will be described using median and quartiles or percentage of the total group as appropriate. All data will be checked for normal distribution. Differences in dependent categorical variables (questionnaire data) will be assessed by McNemar test. Differences in continuous variables over time (all other variables) will be assessed by ANOVA or Friedman tests, depending on the distribution of the data. Bonferroni correction for multiple testing will be used. Pearson or Spearman correlation, depending on the distribution of data, will be used to assess correlation between variables (blood alcohol, gut permeability, bacterial translocation, neutrophil function, oxidative stress). All tests will be performed on a 5% significance level.

# Work and Time Plan

| Months  Task | 1-3 | 4-6 | 7-9 | 10-12 |
| --- | --- | --- | --- | --- |
| Study initiation, database development | x |  |  |  |
| Patient recruitment and clinical study | x | x |  |  |
| Sample batch analysis |  |  | x | x |
| Biostatistical analysis |  |  |  | x |
| Evaluation of experiments;  data preparation for publication |  |  |  | x |
| Data presentation on scientific meetings |  |  |  | x |
| Preparation of follow-up study |  |  |  | x |

# Ethical considerations

The study participants are healthy volunteers. They will get a compensation of 100 € for completing all study visits to cover their expenses due to the participation in this study. Binge drinking can potentially be harmful, therefore the dose of alcohol chosen is at the lowest limit to fulfil the definition of binge drinking. Furthermore, several safety measures will be applied: Patients will be monitored until they recover; they are not allowed to drive a car on the day of the study and will therefore receive taxi vouchers or need to use public transport and/or need to be accompanied by an adult friend or family member on their way home.

Participants with not benefit directly for the participation in this study. Participants will benefit indirectly from a detailed assessment of their health status. As a further benefit the Principal Investigator of the study will inform each participant individually on the medical risks of binge drinking at visit 2. Taken together in our view the risks and benefits of the study are balanced.

The protocol, informed consent form and the participant information sheet have been submitted to the Ethics Committee of the Medical University of Graz, Austria.

The Investigator will obtain approval from the Ethics Committee of the Medical University of Graz, Austria for all amendments to the original approved documents.

# Quality control

The study will be performed in accordance to the ICH-GCP guidelines.

# Insurance

All participants will be insured by the study insurance of the Medical University of Graz.

# Responsibilities

The Principal Investigator is accountable for the conduct of the study. If any responsibilities are delegated, the Principal Investigator should maintain a list of appropriately qualified persons to whom he/she delegated specified significant study-related duties

# References

1. Mathurin P, Deltenre P. Effect of binge drinking on the liver: an alarming public health issue? Gut. 2009;58(5):613-7. doi:10.1136/gut.2007.145573.

2. Connell AM, Patton E, McKillop H. Binge Drinking, Depression, and Electrocortical Responses to Emotional Images. Psychology of addictive behaviors : journal of the Society of Psychologists in Addictive Behaviors. 2015. doi:10.1037/adb0000071.

3. O'Connor RM, Kenny PJ. Neuroscience: Binge drinking and brain stress systems. Nature. 2015;520(7546):168-9. doi:10.1038/520168a.

4. Mason-Jones AJ, Cabieses B. Alcohol, binge drinking and associated mental health problems in young urban chileans. PloS one. 2015;10(4):e0121116. doi:10.1371/journal.pone.0121116.

5. Keshavarzian A, Farhadi A, Forsyth CB, Rangan J, Jakate S, Shaikh M et al. Evidence that chronic alcohol exposure promotes intestinal oxidative stress, intestinal hyperpermeability and endotoxemia prior to development of alcoholic steatohepatitis in rats. Journal of hepatology. 2009;50(3):538-47. doi:10.1016/j.jhep.2008.10.028.

6. Hartmann P, Chen WC, Schnabl B. The intestinal microbiome and the leaky gut as therapeutic targets in alcoholic liver disease. Front Physiol. 2012;3:402. doi:10.3389/fphys.2012.00402.

7. Mutlu EA, Gillevet PM, Rangwala H, Sikaroodi M, Naqvi A, Engen PA et al. Colonic microbiome is altered in alcoholism. American journal of physiology Gastrointestinal and liver physiology. 2012;302(9):G966-78. doi:10.1152/ajpgi.00380.2011.

8. Bala S, Marcos M, Gattu A, Catalano D, Szabo G. Acute binge drinking increases serum endotoxin and bacterial DNA levels in healthy individuals. PloS one. 2014;9(5):e96864. doi:10.1371/journal.pone.0096864.

9. Louvet A, Mathurin P. Alcoholic liver disease: mechanisms of injury and targeted treatment. Nature reviews Gastroenterology & hepatology. 2015;12(4):231-42. doi:10.1038/nrgastro.2015.35.

10. Szabo G. Gut-liver axis in alcoholic liver disease. Gastroenterology. 2015;148(1):30-6. doi:10.1053/j.gastro.2014.10.042.
